# Distal extrahepatic cholangiocarcinoma mimicking groove pancreatitis: A case report and literature review

**DOI:** 10.3389/fonc.2022.948799

**Published:** 2022-09-27

**Authors:** Chaoqun Han, Xin Ling, Liping Sheng, Ming Yang, Rong Lin, Zhen Ding

**Affiliations:** ^1^Division of Gastroenterology, Union Hospital, Tongji Medical College, Huazhong University of Science and Technology, Wuhan, China; ^2^Division of Pathology, Union Hospital, Tongji Medical College, Huazhong University of Science and Technology, Wuhan, China

**Keywords:** cholangiocarcinoma, groove pancreatitis, obstructive jaundice, ERCP, EUS

## Abstract

**Background:**

The differential diagnosis between cholangiocarcinoma and groove pancreatitis is quite challenging. Groove pancreatitis is commonly misdiagnosed as periampullary tumors. We reported a case of distal extrahepatic cholangiocarcinoma mimicking groove pancreatitis.

**Case report:**

A 57-year-old male patient was transferred to our hospital after endoscopic retrograde cholangiopancreatography (ERCP) with stent placement in the common bile duct due to obstructive jaundice at a local hospital. Groove pancreatitis was considered based on the clinical manifestations and multiple examinations [including computed tomography (CT), magnetic resonance cholangiopancreatography (MRCP), and endoscopic ultrasonography (EUS)]. The patient’s symptoms and laboratory results almost returned to normal after conservative treatments. Interestingly, his symptoms and laboratory results worsened after the stent was removed. We performed a second EUS process and found a lesion in the lower common bile duct. Finally, the patient underwent pancreatoduodenectomy, and the diagnosis was confirmed as moderately differentiated adenocarcinoma of the common bile duct.

**Conclusion:**

Our case highlights the fact that distal extrahepatic cholangiocarcinoma, which is a malignant disease, can mimic a benign condition like groove pancreatitis. Our case also raises the concern that performing stent placement through ERCP to relieve jaundice without a clear diagnosis could interfere with further evaluation of the disease.

## Introduction

Cholangiocarcinoma (CCA) is a relatively rare malignant disease that originates from the epithelium of bile ducts. Jaundice and abdominal pain are the most frequent symptoms. Groove pancreatitis is an uncommon form of chronic pancreatitis involving the area between the pancreatic head, duodenum, and common bile duct. The symptoms mainly include chronic abdominal pain, vomiting, and weight loss. Due to the specific pathogenic site, groove pancreatitis poses diagnostic challenges and should be differentiated from other periampullary diseases mainly including pancreatic and biliary carcinomas (1). Published case reports associated with the differential diagnosis between cholangiocarcinoma and groove pancreatitis are limited. Herein, we reported a case of distal extrahepatic cholangiocarcinoma (dCCA) mimicking groove pancreatitis.

## Case report

A 57-year-old man who suffered from upper abdominal pain for over 1 year and worsening for 2 months was admitted to a local hospital initially. He had progressive xanthoderma and lost 4 kg of weight in 2 months. This patient had a previous history of recurrent duodenal ulcer and alcohol abuse for over 30 years. Physical examination suggested right-upper abdominal pain and jaundice. The laboratory results of the local hospital showed a significant increase in the serum levels of carbohydrate antigen 19-9 (CA19-9; 160.3 U/L), total bilirubin (TBIL; 103.9 μmol/L), γ-glutamyl transpeptidase (γ-GT; 1,341 U/L), alkaline phosphatase (ALP; 687 U/L), alanine aminotransferase (ALT; 184 U/L), and aspartate aminotransferase (AST; 69 U/L). A rough patch of mucosa was found in the descending duodenal region through gastroscopy, and the following biopsy suggested chronic inflammation. In order to relieve the symptoms of jaundice, the patient underwent endoscopic retrograde cholangiopancreatography (ERCP) with stent deployment in the common bile duct at a local hospital.

**Figure 1:**
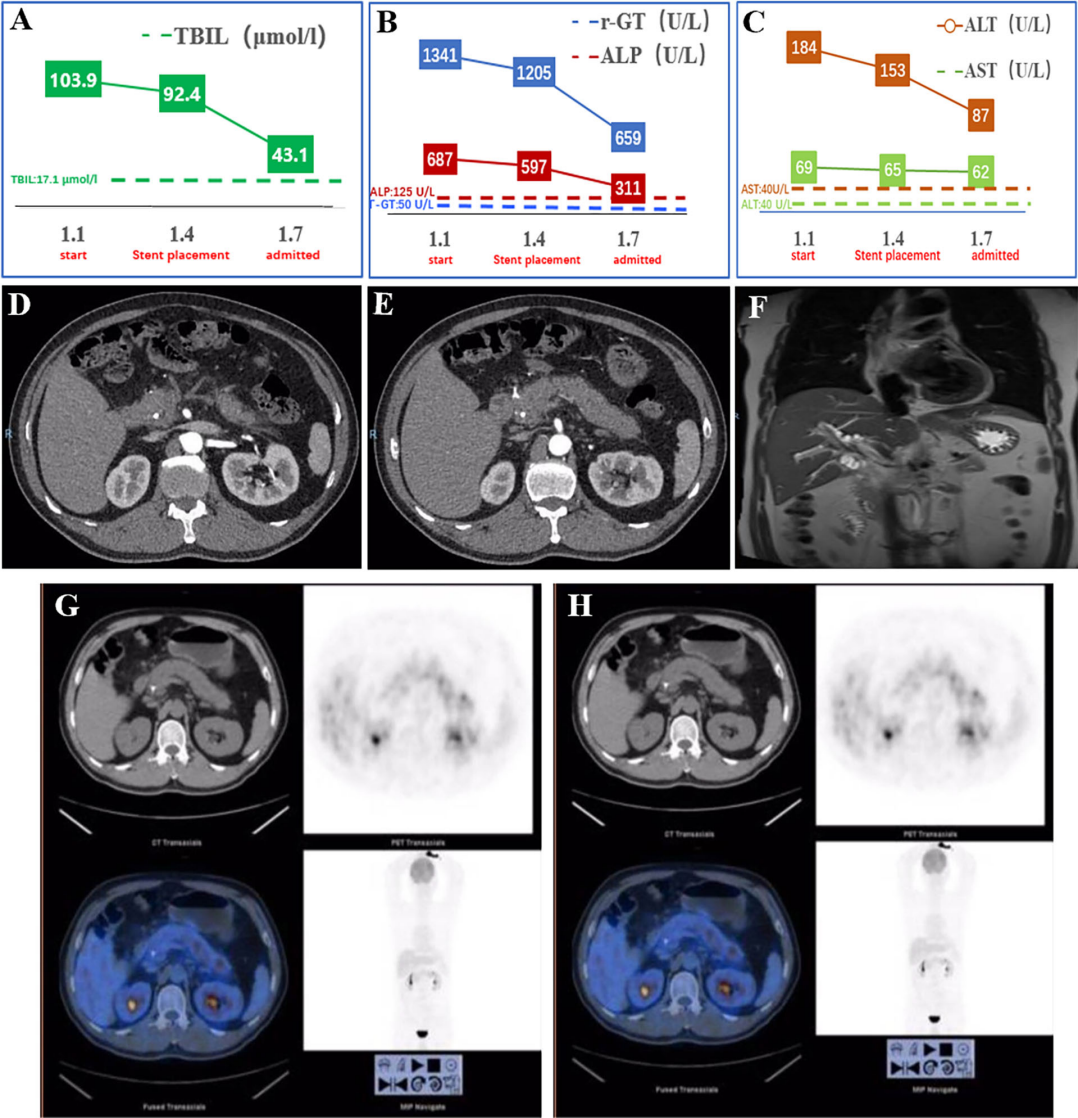
Examination results before and on admission. **(A–C)** The TBIL, γ-GT, ALP, AST, and ALT changing curve of the patient. The measuring frequency was every 3 days in the local hospital. **(D, E)** The abdominal and contrast-enhanced CT scan showed a hyperdense mass in the common bile duct (biliary stent). The pancreatic parenchyma is plump, and adipose tissue around the pancreas is cloudy, which suggested pancreatitis. **(F)** MRCP showed stenosis of the common bile duct. **(G, H)** PET-CT did not show any signs of malignancy. TBIL, total bilirubin; γ-GT, γ-glutamyl transpeptidase; ALP, alkaline phosphatase; AST, aspartate aminotransferase; ALT, alanine aminotransferase.

**Figure 2:**
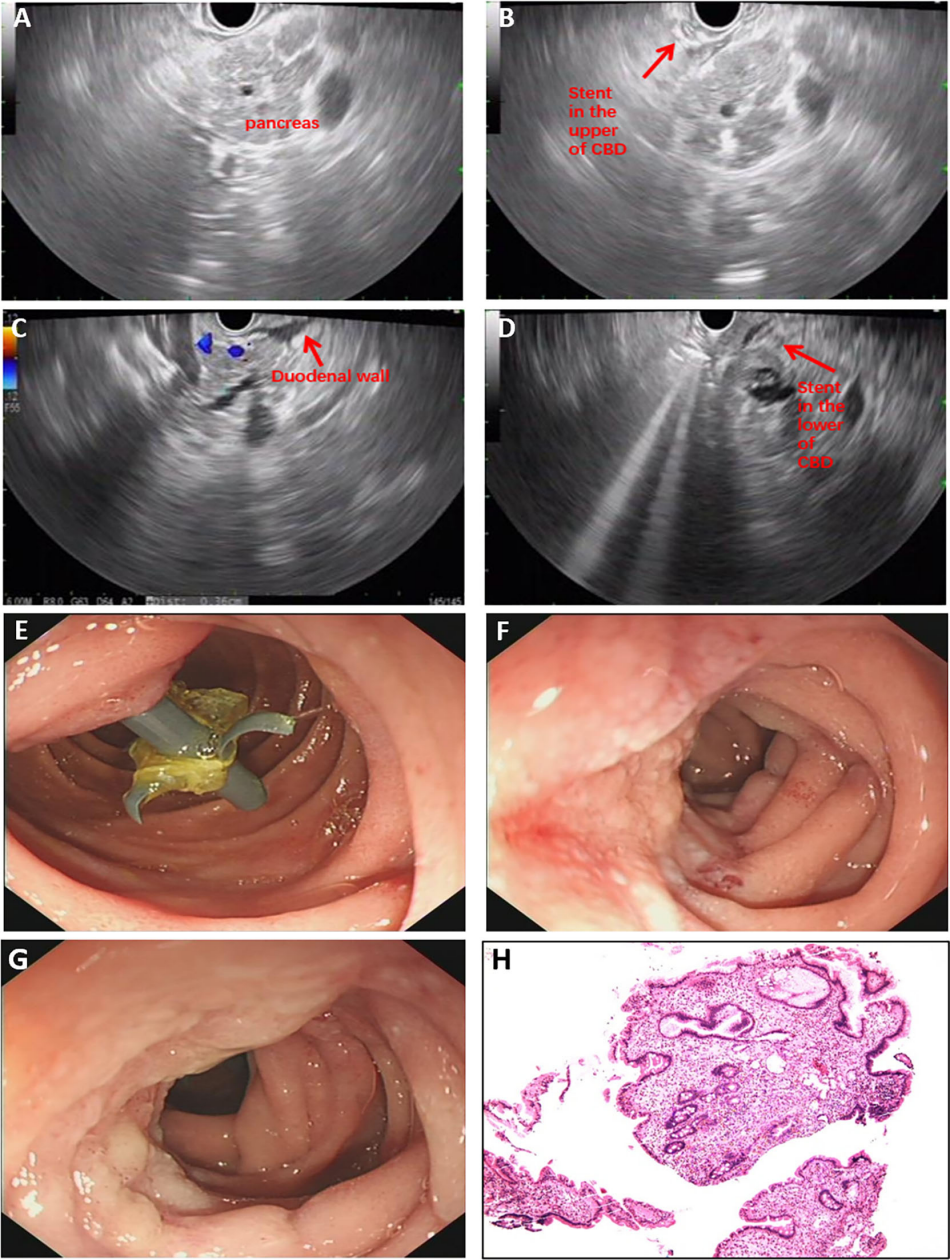
Examination results after admission. **(A–D)** The EUS scan showed the stent in the common bile duct and inhomogeneous echo of pancreatic parenchyma. **(E–H)** Gastroscopy found the rough mucosa of descending duodenum region and the remaining stent, but biopsy suggested inflammation. EUS, endoscopic ultrasonography.

He was transferred to our hospital for further evaluation and treatment. All the clinical indicators of laboratory results were better on his admission compared to the results before stent deployment (**Figures 1A–C**). A week later, the serum levels of TBIL, γ-GT, ALP, ALT, and AST gradually decreased. In order to make a definite diagnosis, we performed further relevant tests. Abdominal computed tomography (CT) indicated a hyperdense mass in the common bile duct, which was the biliary stent. The pancreatic parenchyma is plump, and the adipose tissue around the pancreas is cloudy, which suggested the possibility of pancreatitis (**Figures 1D, E**). Magnetic resonance cholangiopancreatography (MRCP) also suggested pancreatitis and stenotic distal common bile duct, but periampullary cholangiocarcinoma was not excluded (**Figure 1F**). Positron emission tomography (PET)-CT did not show any signs of malignant lesions (**Figures 1G, H**). The remaining stent in the common bile duct and an inhomogeneous echo of pancreatic parenchyma were seen through endoscopic ultrasonography (EUS) (**Figures 2A–D**). However, the biopsy of the rough mucosa in the descending duodenal region still suggested inflammation (**Figures 2E–H**).

Finally, we suspected the diagnosis to be groove pancreatitis based on the above clinical information (**Table 1**). The patient’s symptoms and results of laboratory investigations almost returned to normal after conservative treatments. We suggested he receive reexamination and have the stent removed after 1 month before he was discharged from the hospital.

**Table 1:** Characteristic comparison between groove pancreatitis and this case.

Groove pancreatitis	This case	Criterion
Mostly men at the age of 40–50 (2)	57-year-old man	**√**
Patients usually have a history of chronic alcohol abuse.	Alcohol abuse over 30 years	**√**
Clinical symptoms include upper abdominal pain, vomiting, and weight loss because of duodenal obstruction.	Upper abdominal pain over 1 year	**√**
Endoscopy shows erosion redness, edema, stenosis, and a polypoid appearance in the descending part of the duodenum.	Gastroscopy revealed a rough patch of mucosa in the descending duodenal region	**√**
Chronic inflammation in the duodenum Scar tissue with fibrosis in the groove area	Biopsy suggested chronic inflammation of mucosa in the descending duodenum	**√**
Amylase and lipase are sometimes slightly elevated (2).	Amylase and lipase were normal.	**×**
ALP and γ-GT could increase in some cases (3).	ALP, 687 U/L; γ-GT, 1341 U/L	**√**
CT indicated that GP is usually observed as a hypodense mass in the groove area. Pancreatic duct and extrahepatic bile duct can be seen to be dilated with a stenotic distal common bile duct.	CT indicated that the pancreatic parenchyma is plump and adipose tissue around the pancreas is cloudy, which suggested pancreatitis. MRCP showed a stenotic distal common bile duct	**√**
Patients could have jaundice when accompanied with stricture of distal common bile duct (3).	Jaundice MRCP showed a stenotic distal common bile duct	**√**
CA19-9 and carcinoembryonic antigen are usually normal but could be elevated in some cases (4).	CA19-9, 160.3 U/L	**√**

ALP, alkaline phosphatase; γ-GT, γ-glutamyl transpeptidase; CA19-9, carbohydrate antigen 19-9; MRCP, magnetic resonance cholangiopancreatography; GP, groove pancreatitis.

**Figure 3:**
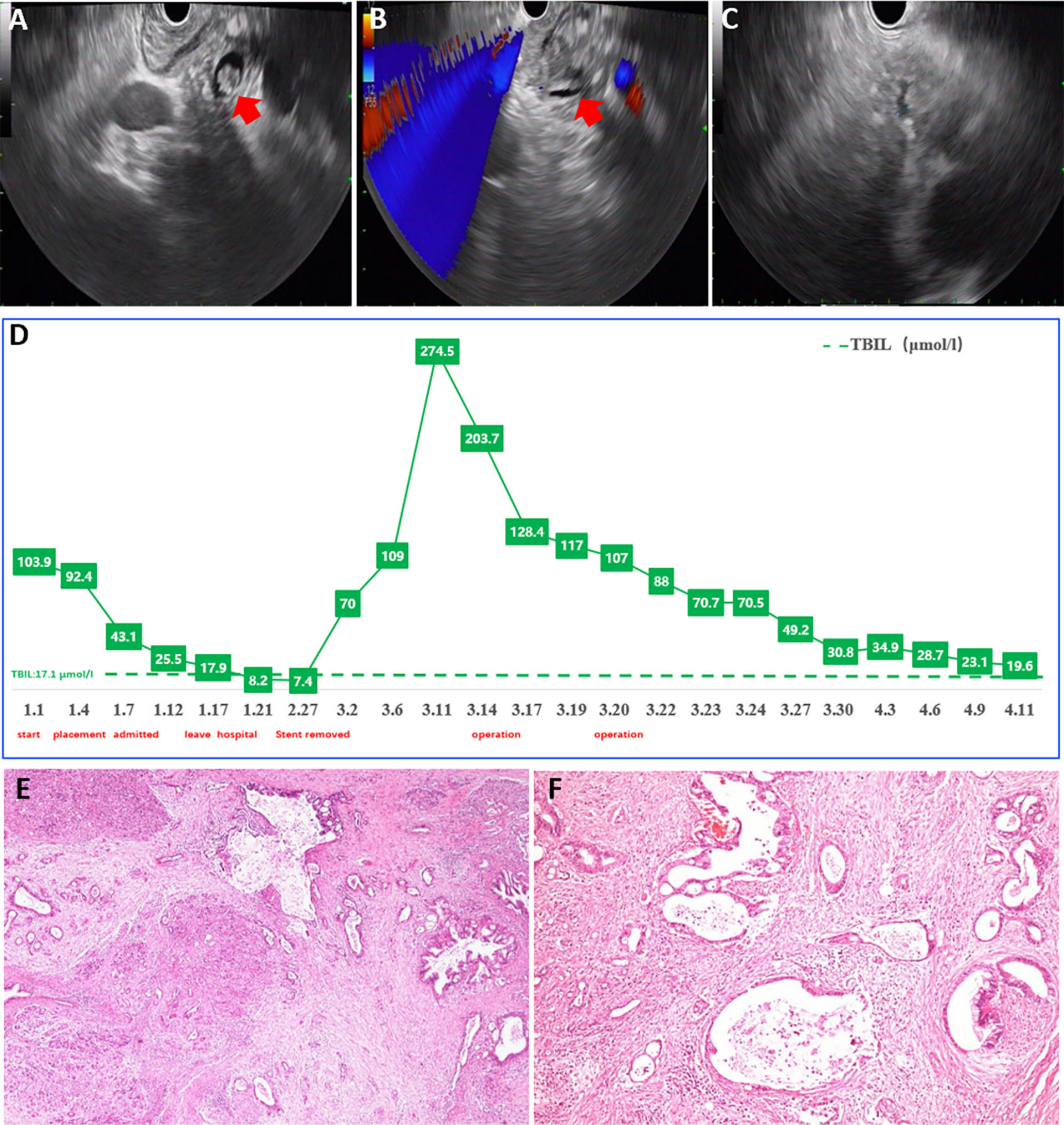
Examination results after readmission. **(A–C)** EUS confirmed a lesion in lower common bile duct. **(D)** The serum level of TBIL after pancreatoduodenectomy. **(E, F)** Pathology evaluation confirmed the diagnosis as moderately differentiated adenocarcinoma of common bile duct. EUS, endoscopic ultrasonography; TBIL, total bilirubin.

One month later, he was admitted to our hospital again. All his laboratory results were normal except for the slight rise of carbohydrate antigen 19-9 (CA19-9). Unexpectedly, symptoms of upper abdominal pain recurred, and the results of laboratory investigations worsened after the stent was removed. Then he underwent the second EUS examination through which a lesion in the lower common bile duct was confirmed (**Figures 3A–C**). For further treatments, he underwent pancreatoduodenectomy and achieved favorable clinical results (**Figure 3D**). Based on the histopathological evaluation of resected specimen, the following diagnosis was confirmed as moderately differentiated adenocarcinoma of the common bile duct (**Figures 3E, F**).

## Discussion

Primary cholangiocarcinoma is a malignant disease that causes obstruction of the extrahepatic bile duct. CCA is difficult to diagnose owing to the silent early clinical manifestations, the low specificity of diagnostic measures, and the lack of absolute diagnostic criteria (5). Clinical, laboratory, endoscopic, and radiologic data aroused the suspicion of CCA, which can be confirmed by biopsy. The normal differential diagnosis for CCA includes pancreatic head carcinoma, other primary liver neoplasms, and metastatic carcinoma (6). Herein, we reported a case of distal extrahepatic cholangiocarcinoma mimicking groove pancreatitis and reviewed the literature associated with the diagnosis of extrahepatic cholangiocarcinoma.

Symptoms and clinical signs associated with CCA should be taken into consideration for diagnosis. The age of incidence most commonly ranges from 50 to 70. There is also a slightly higher incidence in men and people of Asian descent (7). When it comes to symptoms, distal cholangiocarcinoma and perihilar cholangiocarcinoma present similarly, manifested as symptoms of cholestasis and cholangitis. Patients with CCA could also have systemic signs of malignancy including weight loss, anorexia, asthenia, and fatigue. The patient in our case showed no specific clinical symptoms. He had jaundice, upper abdominal pain, weight loss, nausea, and vomiting. Predilection age and alcohol abuse are both associated with CCA and groove pancreatitis (2, 8). Laboratory analysis is mostly unspecific for CCA because it typically reflects cholestasis and cholangitis. The investigations of our case showed almost the same results as groove pancreatitis except for the normal serum levels of amylase and lipase, which could slightly elevate in groove pancreatitis. Serum levels of CA19-9 could work as evidence for the diagnosis of CCA. Interestingly, elevated CA19-9 can also be observed in groove pancreatitis (4), which misled us in our case.

Imaging evaluation plays a crucial role in the management of CCA in terms of diagnosis and response to treatments. The radiologic modalities used for suspected CCA include CT, MRI/MRCP, EUS, and PET-CT. CT is widely considered the standard imaging modality, as it is non-invasive and has the advantage of evaluating extrabiliary involvement. The accuracy of CT in the detection of portal vein involvement and arterial involvement reported as 87% and 93% (9) is fairly high. MRI/MRCP is currently the preferred imaging method because of the advantage of improving the conspicuity of the biliary tree and intrabiliary lesions (10). It has high accuracy to evaluate prognosis (11). Its accuracy in assessing tumor extent and resectability is as high as 96% and 94%, respectively (12, 13). The role of PET-CT in the diagnosis of CCA is somewhat controversial due to the possible false-positive (in terms of inflammation) or false-negative results (in terms of mucinous tumors) (14, 15). Nevertheless, PET-CT shows quite accurate and specific ability in detecting nodal metastases and distant metastases (16, 17). EUS has emerged as an important method for the diagnosis of CCA. It has high sensitivity in detecting extrahepatic lesions and more distant tumors, with the additional ability of helping to judge the resectability of tumors (18). In our case, no specific lesions suspected as CCA were found, but there were signs of pancreatitis accompanied by a rough patch of mucosa in the descending duodenal area and stenotic distal common bile duct. These imaging figures highly suggested groove pancreatitis, confusing our diagnosis.

Biopsy with cytologic and histological analyses are the gold standard for the diagnosis of CCA. Invasive tests, such as ERCP (*via* brushings), could provide access to targeted lesions for cytology samples. However, brushings alone may result in low diagnostic yield due to a limited number of cells (5). As the additional test for samples, fluorescence *in situ* hybridization (FISH) combined with brush cytology could raise the sensitivity (35%–60%) (19, 20). EUS–fine-needle aspiration (EUS-FNA) provides another option for biopsy with less trauma and higher precision. According to a meta-analysis, EUS-FNA shows a higher diagnostic yield than ERCP (75% *vs* 49%) (21). However, EUS-FNA is not encouraged in some specific cases due to the concern of tumor spread, especially in terms of patients with perihilar cholangiocarcinoma. Immunohistochemical (IHC) staining is helpful for the diagnosis and differential diagnosis of CCA, as well as the prediction of prognosis. CCA are positive for cytokeratin (CK) 7 and CK19, negative for caudal-type homeobox transcription factor (CDX) 2, and generally negative for CK20 (22). These markers can help to improve the accuracy of distinguishing metastatic tumors such as metastatic colorectal cancer. However, there are currently no efficient markers to differentiate CCA from pancreatic adenocarcinoma and other upper gastrointestinal neoplasms (6). In our case, we focused on the lesion of descending duodenal region. The following biopsy suggested inflammation, which further misled our judgment.

Later, we reflected on this unique case. As noted, the clinical presentations and laboratory investigations were strikingly similar to those of groove pancreatitis. Moreover, the patient happened to have a rough patch of mucosa in the descending duodenal region with negative biopsy results, which misled the diagnosis. The other interesting part of our case was that the patient underwent ERCP with plastic stent deployment in the common bile duct to relieve jaundice without a clear diagnosis at a local hospital. EUS showed no malignant signs before the stent was removed. We presumed that the placement of a stent before further examinations is a major cause of the confusing imaging features of this patient. It is reported that the presence of a biliary stent could decrease the sensitivity of EUS in detecting CCA (70% *vs* 90%) (23). Although the biliary stent may serve as a mark of the bile duct, its acoustic shadow and the thickened and asymmetrical bile duct wall that resulted from it could interfere with the evaluation of EUS (24). However, there is limited research associated with the impact of an indwelling stent on the evaluation of biliary and pancreatic tumors, which should be evaluated in the future. All in all, our case reminded us to choose the appropriate moment to perform ERCP to deal with obstruction jaundice. Stent placement needs to be performed cautiously before a patient is diagnosed.

Groove pancreatitis is a rare form of chronic pancreatitis. Most patients are male with a history of alcohol addiction. Obstructive jaundice could be observed in a large number of patients with alcohol addiction and stenotic common bile duct (25). Imaging evaluations commonly show thickening of the duodenal wall, augmented head of the pancreas, and stenosis of the common bile duct. In view of the above manifestations, the differential diagnosis of groove pancreatitis with periampullary tumors is challenging. We have noticed several cases where groove pancreatitis is misdiagnosed as pancreatic or duodenal carcinoma (26, 27), while bile duct carcinoma rarely mimics groove pancreatitis. There are some imaging features that may contribute to tell the differences between groove pancreatitis with neoplastic lesions. Groove pancreatitis usually presents with marked duodenal wall thickening, cystic lesion of the groove area, and smoothly, instead of irregularly, tapered stricture of the common bile duct (28). It is worth mentioning that we believe that EUS may serve as an important method in the differential diagnosis of CCA and groove pancreatitis. As for groove pancreatitis, the EUS features present with a hypoechoic mass between the duodenal wall and the pancreatic parenchyma, narrowing of the duodenal lumen, and stricture of the common bile duct and/or pancreatic duct (29). As for cholangiocarcinoma, a bile duct mass, usually hypoechoic or heterogeneous, can be seen. A review of EUS may confirm an inhomogeneous echo of the lesion in the lower common bile duct (30).

## Conclusion

Our case highlights the fact that distal extrahepatic cholangiocarcinoma, which is a malignant disease with a poor prognosis, can mimic a benign condition like groove pancreatitis. The differential diagnosis between dCCA and groove pancreatitis is challenging but important for subsequent treatments. Our case also raises the concern that performing stent placement through ERCP to relieve jaundice without a clear diagnosis could interfere with further evaluation of the disease. Hence, clinicians should consider comprehensively in terms of the hard diagnosis of ampullary diseases.

## Data availability statement

The original contributions presented in the study are included in the article/supplementary material. Further inquiries can be directed to the corresponding authors.

## Ethics statement

This study was reviewed and approved by Ethics Committee of Tongji Medical College, Huazhong University of Science and Technology. The patients/participants provided their written informed consent to participate in this study.

## Author contributions

CH and XL performed the literature search, data extraction, and drafting of the manuscript. LS helped with data collection. MY provided pathologic findings. RL and ZD designed the study and edited the manuscript as the corresponding authors. All authors contributed to the article and approved the submitted version.

## Funding

This study was supported in part by the National Natural Science Foundation of China (Nos. 82170570, 81800467).

## Conflict of interest

The authors declare that the research was conducted in the absence of any commercial or financial relationships that could be construed as a potential conflict of interest.

## Publisher’s note

All claims expressed in this article are solely those of the authors and do not necessarily represent those of their affiliated organizations, or those of the publisher, the editors and the reviewers. Any product that may be evaluated in this article, or claim that may be made by its manufacturer, is not guaranteed or endorsed by the publisher.
